# A new implicit review instrument for measuring quality of care delivered to pediatric patients in the emergency department

**DOI:** 10.1186/1471-227X-7-13

**Published:** 2007-08-23

**Authors:** Madan Dharmar, James P Marcin, Nathan Kuppermann, Emily R Andrada, Stacey Cole, Danielle J Harvey, Patrick S Romano

**Affiliations:** 1 Department of Pediatrics, University of California, Davis, USA; 2 Center for Health Services Research in Primary Care, University of California, Davis, USA; 3 Department of Emergency Medicine, University of California, Davis, USA; 4 Department of Public Health Sciences, University of California, Davis, USA; 5 Division of General Internal Medicine; University of California, Davis, USA

## Abstract

**Background:**

There are few outcomes experienced by children receiving care in the Emergency Department (ED) that are amenable to measuring for the purposes of assessing of quality of care. The purpose of this study was to develop, test, and validate a new implicit review instrument that measures quality of care delivered to children in EDs.

**Methods:**

We developed a 7-point structured implicit review instrument that encompasses four aspects of care, including the physician's initial data gathering, integration of information and development of appropriate diagnoses; initial treatment plan and orders; and plan for disposition and follow-up. Two pediatric emergency medicine physicians applied the 5-item instrument to children presenting in the highest triage category to four rural EDs, and we assessed the reliability of the average summary scores (possible range of 5–35) across the two reviewers using standard measures. We also validated the instrument by comparing this mean summary score between those with and without medication errors (ascertained independently by two pharmacists) using a two-sample t-test.

**Results:**

We reviewed the medical records of 178 pediatric patients for the study. The mean and median summary score for this cohort of patients were 27.4 and 28.5, respectively. Internal consistency was high (Cronbach's alpha of 0.92 and 0.89). All items showed a significant (p < 0.005) positive correlation between reviewers using the Spearman rank correlation (range 0.24 to 0.39). Exact agreement on individual items between reviewers ranged from 70.2% to 85.4%. The Intra-class Correlation Coefficient for the mean of the total summary score across the two reviewers was 0.65. The validity of the instrument was supported by the finding of a higher score for children without medication errors compared to those with medication errors which trended toward significance (mean score = 28.5 vs. 26.0, p = 0.076).

**Conclusion:**

The instrument we developed to measure quality of care provided to children in the ED has high internal consistency, fair to good inter-rater reliability and inter-rater correlation, and high content validity. The validity of the instrument is supported by the fact that the instrument's average summary score was lower in the presence of medication errors, which trended towards statistical significance.

## Background

According to Donabedian's quality of care model [1], the structure in which care is delivered influences the process of care, which in turn influences patient outcomes. Although outcomes represent the most important measure of health system performance, adverse outcomes such as mortality may not occur often enough to provide useful information about quality of care in emergency departments (EDs). This is particularly true when evaluating outcomes in Pediatric EDs. Other outcome measures, including appropriateness of admission and return visits within 24 hours of ED discharge [2-5], may not be sufficiently sensitive to the process of care within the ED because of confounding factors, such as access to post-ED primary care. Therefore, more sensitive instruments are needed to assess the processes of care provided in EDs, in particular for children. The development of such instruments will facilitate the evaluation of new interventions to improve the quality of emergency care, and enable clinicians to quantify and take appropriate steps to rectify deficiencies in emergency services.

Peer review plays an important role in the ascertainment of quality of care [6-13] both at the individual provider level [6,8,9,14,15] and at the system level [6,10-13]. Implicit review is a type of peer review where assessments of quality are based on expert reviewers' judgment of care [1]. Structured review of medical records to assess the "implicit" quality of care has been shown to have high face validity [8] and offers better inter-rater reliability [7,8] than unstructured review [7].

The purpose of this study was to develop, test, and validate an implicit review instrument that measures quality of care delivered to children receiving care in EDs, based on physician review of medical records. Our specific goals were: 1) to apply our implicit review instrument to measure quality of care for acutely ill and injured children presenting to four rural EDs, 2) to assess the reliability of this instrument using standard tools and measures, and 3) to validate the instrument using an explicit measure, namely, the occurrence of medication errors, as well as against a separate reviewer's assessment of quality.

## Methods

### Design of the study

As part of a larger study designed to evaluate ways of improving care of critically ill children in rural EDs in California, we developed an instrument to measure the quality of care provided to pediatric patients in EDs. We applied our instruments to a consecutive sample of pediatric patients using retrospective chart review, and validated the instrument using standard instrument assessment and validation techniques.

### Setting

Because the primary purpose of our study was to measure and improve the quality of care provided to children in rural EDs, we selected four EDs located in designated rural areas as defined by California's Office of Statewide Health Planning and Development [16] and the Federal Center for Medicare and Medicaid Services [17]. All of these EDs are also located in "underserved" communities, according to the Health Resources and Services Administration's definitions of Health Professional Shortage Areas, Medically Underserved Areas and Medically Underserved Populations [18]. The number of children treated in the EDs ranges from 2,200 to 7,500 annually, and the treating physicians include some trained in emergency medicine and some trained in other specialties.

### Selection of Participants

We included all children older than two days but younger than 17 years of age who presented to one of the participating EDs between January 1, 2000 and June 30, 2003, if they were triaged at the highest of three acuity levels. We chose to assess quality among the most ill patients because we wanted to focus our measurement and our interventions on patients who are at highest risk of an adverse event and would likely benefit the most from receiving high-quality care in the ED. All four of the participating EDs used a three-level triage system, with almost identical definitions for the highest acuity level. Some examples of clinical presentations triaged at the highest acuity level include respiratory distress with hypoxia, status epilepticus, and trauma associated with a Glasgow Coma Scale of less than 13. We identified patients by reviewing ED paper or computer logbooks, which included information on each patient's age, diagnoses, and triage level.

### Structured Implicit Review Instrument

We developed an implicit review instrument, the "Pediatric Emergency Department Quality Assessment Scale," according to guidelines published by Rubin, et al [19]. We developed our instrument by modifying instruments previously published and validated by the RAND Corporation, including the RAND PRO Quality Review Validation Study (PQRVS) form and the DRG/QC Study Implicit Review form [11,20-22]. Modifications were made to make the instrument applicable to patients receiving care in the ED. The instrument (Additional file 1) was structured by encompassing four defined aspects of physician care in the ED, including initial data gathering about acute problems; integration of information and development of appropriate diagnoses; the initial treatment plan and orders; and the plan for disposition and follow-up. We included an additional question to assess the global quality of care provided to the patient during his or her stay in the ED [11]. These assessments were measured on a 7-point ordered adjectival scale representing appropriateness of care (extremely inappropriate; very inappropriate; somewhat inappropriate; intermediate; somewhat appropriate; very appropriate; extremely appropriate) [11]. The four defined aspects of physician care in the ED and the global quality question described above formed the five items of the instrument. We summed the scores on these items to generate an overall summary score for each ED encounter. There was no training period in which sample cases were evaluated together to anchor high and low scores. Quality of care based on an individual item was considered "acceptable" if the rating was "somewhat appropriate," "very appropriate," or "extremely appropriate" and "unacceptable" if the rating was "extremely inappropriate," "very inappropriate," "somewhat inappropriate," or "intermediate." Quality of care for the summary score was considered "acceptable" if the summary score was greater than 20 (summary score range of 5–35).

In addition, we further asked a question that was used to assess the construct validity of the instrument after reviewing each individual patient chart. Reviewers were asked, "What is the likelihood that you would want this physician taking care of your child in the Emergency Department?" [10,11] This question, referred to as the "validation question," was measured on a 7 point ordered adjectival scale from extremely unlikely to extremely likely.

### Ascertainment of medication errors

As the primary means of assessing validity, we independently ascertained medication errors that occurred during each visit to the ED. Two pediatric pharmacists reviewed the de-identified medical records and the frequency of medication errors was determined using previously published guidelines detailed below [23,24]. None of the hospitals has a computerized medication order entry system; none uses software to verify dosing, interval, or administration technique, or has a verification system for checking allergies or contraindications to medications.

We explicitly evaluated all medications ordered and/or dispensed in the ED. We identified medication errors as outlined by the National Coordinating Council for Medication Error Reporting [25]. For the purposes of validating the implicit review instrument, we selected medication errors that were identified as physician related (i.e., incorrect dosage, dosage form, dosage frequency, appropriateness of medication, and contraindications for medication, such as allergies or drug interactions) [23,24]. Some other types of errors identified by Bates and Kushal were included (e.g., inappropriate frequency), whereas others were either not applicable to the ED setting or not ascertainable by retrospective chart review (e.g., wrong date, wrong patient). When there were disagreements as to whether or not a medication error occurred, both pharmacists, in the presence of a pediatrician, developed a consensus determination.

### Data Collection and Processing

For the purposes of describing the population studied, a research assistant abstracted patient demographic data, diagnostic data, and disposition of care data from each medical record. He or she then copied each record, blacking-out all hospital and patient identifiers so that physician reviewers would be blinded to the identity of the hospital and the physician. Two pediatricians board-certified in Pediatric Emergency Medicine (PEM) and with more than 5 years of experience in PEM reviewed the de-identified medical records, blinded to hospital, provider and patient information, and to each other's assessments. We did not provide explicit instructions on how to score individual items on the instrument to the reviewers. If there was a disagreement in the quality ranking greater than 2 points on the ordered adjectival scale, cases were re-examined and discussed by both physicians together, who then were allowed to change their scoring after discussion [9,13,15]. All analyses for reliability were conducted prior to the physicians' re-examination of discrepant scores.

### Primary Data Analysis

We performed the statistical analyses using SAS software version 8.2. Reliability analyses were conducted for each item of the tool as well as for the total summary score. Because we anticipate the instrument will be used by at least two reviewers, validation analyses focused on the average of the two reviewer's total summary score. Because some limitations have been noted in the use of implicit review to measure quality [7,8,15], we sought to maximize reliability [9,13,15] by averaging item-specific scores from the two reviewers. We assessed the quality of care instrument using measures of internal consistency, inter-rater reliability and construct validity. Internal consistency was assessed for each of the individual reviewers' scores using the Cronbach's alpha coefficient, item-total correlations, and the change in Cronbach's alpha after removal of each single item from the tool. Inter-rater reliability was assessed on an item-specific basis using the Spearman rank correlation coefficient to determine if subjects were ranked similarly by both reviewers. Inter-rater reliability was also measured using the Intraclass Correlation Coefficient (ICC) [26,27], calculated as described by Shrout and Fleiss [26]. Because the purpose of the instrument is to compare the quality of care among different cohorts of patients, we relied on the ICC as the primary measure of reliability [26]. That is, we consider it more important that different raters rank patients' quality of care in a similar order than that they assign identical numerical ratings on the adjectival scale. If two reviewers score the quality of care that patients receive in a similar rank order, even though one reviewer may be an "easier grader" with a higher mean score, the ICC would be high, whereas other methods used to measure agreement such as the Kappa statistic, do not take into account the systematic difference in the ratings. The measure of inter-rater agreement was determined based on the categorization recommended by Landis and Koch [28].

We assessed the validity of the implicit quality review instrument using two methods. First, we compared the mean total summary score for patients with and without physician-related medication errors using the two-sample t-test. Due to the mild skewness of the data, we also used the non-parametric Mann-Whitney test. We also assessed the construct validity of the instrument by analyzing the correlation between one reviewer's total summary score and the other reviewer's "validation question" score, as well as the correlation between both reviewers' mean summary score and their mean "validation question" score, using the Spearman rank correlation coefficient.

### Human Subjects

This research study was approved by the Human Subjects Review Committee at the University of California, Davis as exempt. Consent from individual patients/guardians was not required.

## Results

During the study period, 20,048 pediatric patients were treated in the four EDs. Of these, 186 patients (0.9%) met entry criteria (that is, were triaged at the highest category). There were 8 (4.3%) charts that either could not be located or did not include the complete ED visit documentation, leaving 178 patients in the final sample treated by a total of 49 physicians. Table 1 describes the patients who were included in the study. The four EDs contributed similar numbers of children and there was an equal distribution of males and females in the overall sample. Most patients were either transferred to a referral hospital for further treatment (42.7%), or were admitted locally for at least 24 hours (19.1%), suggesting that most of these patients seeking care in the ED were appropriately triaged into the highest triage category. The reviewer's took approximately 15 minutes to apply the instrument to each medical record. The total summary score for this cohort ranged between 14 – 35, with a mean of 27.4 (SD = 4.1), and measure of skewness of -0.869. The percentage of ratings at the upper limit of the scale for the total summary score (35) by at least one of the reviewers was 10.7% (n = 19) and 0.005% (n = 1) for both reviewers. The mean total summary score for the four hospital settings, A-D, were 28.3, 23.3, 28.6 and 27.8, respectively.

**Table 1:** Baseline patient characteristics (N= 178)

**Patient characteristics**	**Frequency (%)**
***Emergency department***	

A	40 (22.5)
B	30 (16.9)
C	57 (32.0)
D	51 (28.7)

***Age (Years)***	

< = 1	28 (15.7)
> 1 – < = 5	49 (27.5)
> 5 – < = 10	39 (21.9)
>10 – < = 16	62 (34.8)

***Gender***	

Male	91 (51.1)
Female	86 (48.3)
Unknown	1 (0.6)

***Disposition of care***	

Admitted	34 (19.1)
Discharged home	24 (13.5)
Admitted for observation for <24 hours	43 (24.2)
Transferred	76 (42.7)
Expired	1 (0.6)

Table 2 shows the percentage of patient encounters judged by the two reviewers to be "acceptable" and "extremely appropriate" for each of the four components of ED care and for the overall quality of care. The overall percentage of acceptable care was high for all of the components, according to both reviewers, ranging from 65.7% – 93.8%. Reviewer 2 judged a greater percentage of the cases to be acceptable than Reviewer 1 for all of the components except for "initial data gathering by physician about acute problems." The percentage of ratings at the upper limit of the scale (score 7) is also shown in Table 2, indicating that the two reviewers differed in their willingness to rate care as "extremely appropriate."

**Table 2:** Frequency of reporting acceptable and extremely appropriate care among the two reviewers

**Implicit review process measure**	Acceptable care*		Extremely appropriate care**	
	***Reviewer 1***	***Reviewer 2***	***Reviewer 1***	***Reviewer 2***

Initial data gathering by physician about acute problems	163 (91.6)	151 (84.8)	12 (6.7)	25 (14.0)
Physician integration of information and development of appropriate diagnoses	147 (82.6)	159 (89.3)	10 (5.6)	41 (23.0)
Physician initial treatment plan and orders	117 (65.7)	126 (70.8)	10 (5.6)	26 (14.6)
Physician plan for disposition and follow-up	146 (82.0)	167 (93.8)	10 (5.6)	64 (36.0)
Overall quality of care	136 (76.4)	141 (79.2)	10 (5.6)	22 (12.4)

* Care ranked as "somewhat appropriate," "very appropriate" and "extremely appropriate"

** Care ranked as "extremely appropriate" (Score 7)

The Cronbach's alpha[29] was calculated for the instrument as shown in Table 3. Internal consistency was very high, with Cronbach's alpha coefficients of 0.92 for Reviewer 1 and 0.89 for Reviewer 2. Both values exceed the recommended threshold value of 0.70 for new instruments [30]. The rating of overall quality of care was the item most highly correlated with the summary score based on the rest of the tool, and this item was associated with the largest drop in the alpha coefficient when it was removed from the instrument.

**Table 3:** Measures of internal consistency

**Deleted variable**	**Reviewer 1**		**Reviewer 2**	
	Item-total correlation*	Cronbach's alpha without item	Item-total correlation*	Cronbach's alpha without item

Initial data gathering by physician about acute problems	0.63	0.94	0.66	0.89
Physician integration of information and development of appropriate diagnoses	0.76	0.91	0.74	0.87
Physician initial treatment plan and orders	0.88	0.89	0.77	0.87
Physician plan for disposition and follow-up	0.83	0.90	0.65	0.89
Assess the overall quality of care provided to the patient	0.94	0.87	0.90	0.83

* Item-total correlation is the correlation between the specified item and the sum of the other four items in the tool.

Cronbach's alpha, Reviewer 1 = 0.92

Cronbach's alpha, Reviewer 2 = 0.89

Table 4 shows inter-rater agreement for each individual item as well as for the total summary score. All items for ED process of care showed a significant (p < 0.005) positive correlation using the Spearman rank correlation coefficient between the two reviewers (0.24 to 0.39). The 5-item summary score had a higher rank correlation at 0.46 (p < 0.0001) than did any of the component items. Each item score was dichotomized as acceptable or unacceptable care to estimate the percent exact agreement. Exact agreement on each item ranged from 70.2% to 85.4%. Table 4 also illustrates the ICC for the average rating of each item as well as for the average summary total score, across the two reviewers. The ICC for the mean total summary score was 0.65. If the instrument were to be used in a setting where only one rater's score was used as the final score, then the ICC would drop to 0.48.

**Table 4:** Inter-rater agreement

**Item**	**Full ordered adjectival scale**	**Dichotomized response (Acceptable vs Unacceptable)**	***Intra-Class Correlation***
	***Spearman rank correlation***	***Percent exact agreement***	***Coefficient***

Initial data gathering by physician about acute problems	0.24	85.4	0.48
Physician integration of information and development of appropriate diagnoses	0.37	79.8	0.57
Physician initial treatment plan and initial orders	0.35	70.2	0.57
Physician plan for disposition and follow-up	0.39	83.7	0.59
Assess the overall quality of care provided to the patient	0.39	75.3	0.60
Total summed score	0.46	86.5	0.65

The instrument was validated by measuring the association between the total summary score and the frequency of medication errors. 129 of patients had medications ordered (either in the ED or at discharge); of these, 34 (26.4%) were found to have a medication error. We found a 19.4% higher incidence of medication errors among children who received "unacceptable" care compared to children who received acceptable care (4/9 [44.4%], versus 30/120 [25.0%]; p = 0.20). The mean total summary scores were lower for children with medication errors (26.5; SD = 4.4) than for children without medication errors (28.0; SD = 4.1), with the difference in means trending towards statistical significance (95% confidence interval: -3.14 to 0.159; p = 0.076). We also used the non-parametric Mann-Whitney test, but results were similar (p = 0.069).

The correlation between Reviewer 1's total summary score and Reviewer 2's "validation question" score was 0.47 (p < 0.0001). The correlation between Reviewer 2's total summary score and Reviewer 1's "validation question" score was 0.36 (p < 0.0001). Finally, the correlation between the mean total summary score (using both reviews) and the mean "validation question" score was 0.95 (p < 0.0001).

## Discussion

In this study, we designed and evaluated an implicit review instrument to assess the quality of care provided to children in the ED. This instrument measures four aspects of care, as well as overall quality of care. When applied by two pediatric emergency medicine physicians to 178 acutely ill and injured pediatric patients seen at four rural EDs, the instrument had high internal consistency reliability and fair to good inter-rater reliability. The validity of the instrument is supported by the fact that the mean total summary score was associated with the incidence of medication errors (an explicit measure). Furthermore, each of the reviewer's total summary score correlated with the other reviewer's "validation question" score (a separate measure of validity), and the mean total summary score was correlated with the mean "validation question" score for the two reviewers.

We also found that in the majority of the visits, the quality of care provided to critically ill pediatric patients in this sample of four rural EDs was considered acceptable by experts in pediatric emergency medicine. This finding that the majority of the care was considered acceptable using implicit and explicit review is similar to previously published reports [10-12,15]. The fact that our instrument has high face and construct validity and fair inter-rater reliability for the individual items and good inter-rater reliability for the total summary score (as measured by ICC) is also consistent with findings of several previous studies using implicit review [7-9,11-15,30]. These findings together suggest a tendency for multiple reviewers to rank quality of care similarly, but not necessarily with the same numerical ratings (e.g., some reviewers tend to assign higher scores than others, but in a similar rank).

With regards to pediatric medication errors, our study identified errors among 26.4% of patients who had medications ordered, which is higher than the previously published range of 5.7% to 14.7% [31-34]. However, most of these latter studies relied on incident report data or voluntary error reports [33,34], which would tend to underestimate actual medication error rates. The medication error rates may also have been higher because of less pediatric experience at the hospitals studied or because the EDs were not all staffed by emergency medicine trained physicians with pediatric experience. Furthermore, our study focused on the most ill pediatric patients presenting emergently to the ED, which would likely tend to increase the prevalence of medication errors in our sample[32].

Peer review plays a central role in many quality assurances strategies [6] both for the evaluation of physician performance as well as program performance [10,22]. The implicit peer review method used in this study has face validity to physicians. Because of the diversity of diagnoses and heterogeneous severity of illness among children presenting to the ED, no explicit measures of quality of care are available that could be applied to a consecutive cohort of unselected ED patients. Implicit review allows assessments to be made that consider the unique characteristics of each patient, taking into account the latest trends and developments of patient management. The structured implicit review approach adopted in this study is designed to capture the strengths of both implicit review (e.g., allowing the reviewer to consider the nuances of the case, which enhances validity) and explicit review (e.g., requiring all reviewers to examine certain elements of care, which enhances reliability) [35].

There are several limitations to our study. First, our instrument was only tested on the most ill pediatric patients presenting to four rural EDs. However, it is for these patients that quality of care is of greatest concern. Second, we only used two reviewers for the assessment of quality of care, which could limit the generalizability of the instrument if other reviewers score charts in a different manner. We recommend further validation of this instrument using more reviewers. The extent to which this instrument is valid and reliable in other settings when applied by other reviewer's and with less ill patients remains requires further study. Third, the ability of our instrument to measure quality is somewhat dependent upon the detail of documentation in the medical record [36]. While the quality of the documentation may affect measurement of the physician's "integration of information," it would be less likely to affect measurement of the physician's "initial data gathering," "initial treatment plan and orders," and "plan for disposition and follow-up," which are documented through orders or laboratory reports as well as physician notes. Fourth, medication errors may have in part influenced the physicians' assessment of quality, making medication errors a less than ideal validation measure. However, many aspects of the review for medication errors could only be appreciated by pharmacist review of pharmacy records, and not by physicians' review of the ED record. Blinding charts of hospital information may not have been completely successful because hospital charts are different; however, this limitation should not affect the reliability or construct validity of the instrument. Finally, despite steps taken to increase inter-rater agreement, our ICC suggests only fair agreement between physician evaluators for individual items on our instrument, but good agreement for the total summary score [28]. We are not discouraged by this finding, however, because we devised the instrument to measure variation in quality of care across different cohorts of patients, expecting that different reviewers may have different overall mean scores. Furthermore, we did not want to artificially increase reviewer agreement by providing *a priori *explicit instructions on how to score individual quality items. Our high Spearman rank correlation suggest that the reviewers tended to rank quality of care similarly, albeit with different mean scores. Previous studies indicate that the reliability of peer review increases with the number of reviewers and hence, using more than two reviewers would probably further increase inter-rater reliability [13,29,37].

By investigating processes of care in EDs and comparing implicit quality of care across sites, it is our goal to better understand the factors that need to be addressed to improve care. Our implicit review instrument could be used to assess whether differences in quality of care exist between different types of EDs, including rural, suburban, urban, or Children's Hospital EDs. Similarly, it could be used to investigate whether the presence of specialty trained or board certified Emergency Medicine physicians is associated with higher quality of care [38-40].

## Conclusion

In conclusion, we have developed a new instrument for the purpose of measuring quality of care among children receiving care in EDs, and we report on the instrument items for a cohort of acutely ill and injured children presenting to rural EDs in Northern California. We expect that this instrument will provide researchers a tool for measuring quality of care in other patient populations, and that this research will lead to future comparisons and investigations aimed at improving the quality of emergency care for children. A future goal would be to validate this instrument in different ED settings with more than two reviewers. For future validation, we would suggest additional outcome measures not directly linked to the quality of care of a particular ED visit, such as readmission or return visits to the ED, or the accuracy of the initial diagnosis.

## Competing interests

The author(s) declare that they have no competing interests.

## Authors' contributions

JPM, PSR, and NK conceived the study, designed the trial, and obtained research funding. JPM, PSR and SLC supervised the conduct of the trial. Data collection was conducted by MD, JPM and SLC. NK and EA refined the implicit review instrument and conducted all chart reviews. SLC and MD managed the data. MD and DJH analyzed the data. MD and JPM drafted the manuscript, and all authors contributed substantially to its revision.

## Pre-publication history

The pre-publication history for this paper can be accessed here:



## Supplementary Material

Additional file 1 Pediatric emergency department quality assessment scale. This file shows the quality assessment instrument. Click here for file
