# Supplementary material for: A new implicit review instrument for measuring quality of care delivered to pediatric patients in the emergency department
Source: BMC Emerg Med. 2007 Aug 23;7:13. doi: 10.1186/1471-227X-7-13 (PMC2000874; doi:10.1186/1471-227X-7-13)
Supplement: Additional file 1 — Pediatric emergency department quality assessment scale. This file shows the quality assessment instrument. [file 1471-227X-7-13-S1.ppt]

## Slide 1
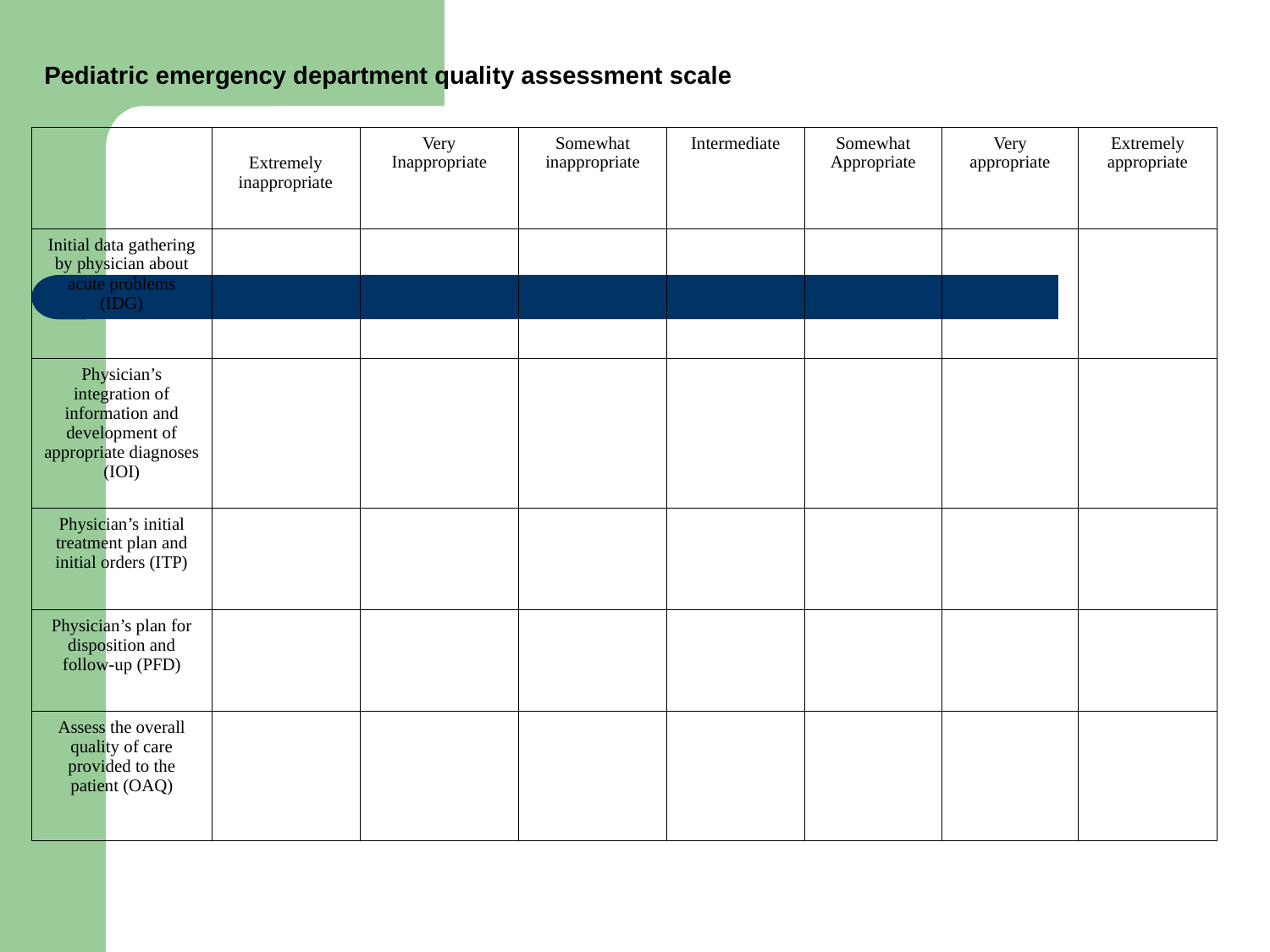

Pediatric emergency department quality assessment scale
| | Extremely inappropriate | Very Inappropriate | Somewhat inappropriate | Intermediate | Somewhat Appropriate | Very appropriate | Extremely appropriate |
| --- | --- | --- | --- | --- | --- | --- | --- |
| Initial data gathering by physician about acute problems (IDG) | | | | | | | |
| Physician’s integration of information and development of appropriate diagnoses (IOI) | | | | | | | |
| Physician’s initial treatment plan and initial orders (ITP) | | | | | | | |
| Physician’s plan for disposition and follow-up (PFD) | | | | | | | |
| Assess the overall quality of care provided to the patient (OAQ) | | | | | | | |
